# Molecular mechanism of Wilms’ tumor (*Wt1*) (+/−KTS) variants promoting proliferation and migration of ovarian epithelial cells by bioinformatics analysis

**DOI:** 10.1186/s13048-023-01124-2

**Published:** 2023-02-24

**Authors:** Xiaomei Wang, Jingyu Zhao, Yixin Zhang, Yuxin Liu, Jinzheng Wang, Ruoxi Shi, Jinxiang Yuan, Kai Meng

**Affiliations:** 1 College of Basic Medicine, Jining Medical University, Jining, China; 2 Collaborative Innovation Center for Birth Defect Research and Transformation of Shandong Province, Jining Medical University, Jining, China; 3 College of Second Clinical Medical, Jining Medical University, Jining, China; 4 Lin He’s Academician Workstation of New Medicine and Clinical Translation, Jining Medical University, Jining, China

**Keywords:** Wilms’ tumor (*Wt1*) (+/−KTS), Ovarian epithelial cells, Proliferation, Migration, Bioinformatics analysis

## Abstract

Epithelial ovarian cancer (EOC) is a gynecological disease with the highest mortality. With the lack of understanding of its pathogenesis, no accurate early diagnosis and screening method has been established for EOC. Studies revealed the multi-faceted function of Wilms’ tumor (*Wt1*) genes in cancer, which may be related to the existence of multiple alternative splices. Our results show that *Wt1* (+KTS) or *Wt1* (−KTS) overexpression can significantly promote the proliferation and migration of human ovarian epithelial cells HOSEpiC, and *Wt1* (+KTS) effects were more evident. To explore the *Wt1* (+/−KTS) variant mechanism in HOSEpiC proliferation and migration and ovarian cancer (OC) occurrence and development, this study explored the differential regulation of *Wt1* (+/−KTS) in HOSEpiC proliferation and migration by transcriptome sequencing. OC-related hub genes were screened by bioinformatics analysis to further explore the differential molecular mechanism of *Wt1* (+/−KTS) in the occurrence of OC. Finally, we found that the regulation of *Wt1* (+/−KTS) variants on the proliferation and migration of HOSEpiC may act through different genes and signaling pathways and screened out key genes and differentially regulated genes that regulate the malignant transformation of ovarian epithelial cells. The implementation of this study will provide new clues for the early diagnosis and precise treatment of OC.

**Supplementary Information:**

The online version contains supplementary material available at 10.1186/s13048-023-01124-2.

## Introduction

Ovarian cancer (OC) is the second most common cause of death from gynecological cancers [1, 2], and its mortality rate ranks first among malignant tumors of the female reproductive tract, which seriously threatens women’s lives and health. Epithelial ovarian cancer (EOC) accounts for > 95% of ovarian malignancies, and its occurrence and development are complex. There is still no accurate early screening method for OC, and most patients are in the advanced stage when receiving treatment. Several previous studies have shown that EOC is mainly derived from the malignant transformation of ovarian surface epithelial cells [3–5]. After the transformation of ovarian epithelial cells, cancer cells typically detach from the superficial basement membrane and rapidly metastasize throughout the peritoneal cavity [6]. Furthermore, some studies have hypothesized that EOC may originate from the fallopian tube [7, 8]. Currently, data on the tubal or ovarian origin of EOC have been conflicting, suggesting that EOC may have a dual origin [9].

EOC is a heterogeneous disease with four main histologic subtypes: serous, clear cell, endometrioid, and mucinous OC, with serous being the most common. Serous OC can be further divided into high-grade serous ovarian cancer and low-grade serous ovarian cancer, which have distinct clinical and molecular features [10]. The high EOC heterogeneity also suggests that it may have different origins. Ovarian epithelial cells were one of the main origins of EOC [9]. The molecular mechanism of malignant transformation of ovarian epithelial cells helps us reveal the process of occurrence and development of ovarian epithelial-derived OC from the source.

Wilms’ tumor gene (*Wt1*) encodes a transcription factor with zinc finger structure, which plays an important role in tumor formation. This had led the National Cancer Institute to list *Wt1* as one of the potential cancer antigens to be targeted by immunotherapy drugs [11]. Pediatric tumor-related studies demonstrated that the *Wt1* gene had been shown to play an important role in renal progenitor differentiation and tumor suppression [12]. The WT1 protein was overexpressed in > 90% of breast cancers, and the 36–38 kDa isoform of WT1 was associated with estrogen receptor deletion in advanced breast cancers [13]. Previous studies of OC had also shown that the *Wt1* gene was highly expressed in EOC tissues and was involved in regulating OC migration and invasion [14, 15]. Our previous study showed that the expression level of *Wt1* in OC tissues was significantly higher than that in normal ovarian tissues, and this gene also played an important regulatory role in OC cell migration, and invasion [16]. Therefore, although the *Wt1* gene was originally described as a classic tumor suppressor gene, the *Wt1* gene may act as a proto-oncogene in many cases.

The *Wt1* gene has 36 different variants, two of which are the most studied, *Wt1* (+KTS) and *Wt1* (−KTS), with differences in three amino acids inserted or excluded between zinc fingers 3 and 4 (lysine, threonine, and serine) [17]. The *Wt1* (+KTS) and *Wt1* (−KTS) variants differed only in three amino acid sequences but had significant functional differences. Studies have shown that *Wt1* (−KTS) induces programmed cell death through transcriptional repression of the Epidermal Growth Factor Receptor (EGFR) gene in osteosarcoma cells [18]. *Wt1* (+KTS) can result in the morphological transition of mammary epithelial cells from an epithelial to a mesenchymal phenotype [19]. Stable expression of *Wt1* (−KTS) in the breast cancer cells significantly suppressed colony formation and cell division, whereas that of *Wt1* (+KTS) did not [20]. We previously investigated the functional roles of the *Wt1* gene and *Wt1* (+/−KTS) variants in granulosa, thecal, and ovarian cortical stromal cells, and found that *Wt1* significantly affects the steroidogenesis process of these cells, whereas the effects of *Wt1* (−KTS) are more obvious [21–23]. Therefore, the *Wt1* gene is a very important regulatory gene in OC, and *Wt1* (+/−KTS) variants may have different regulatory roles.

In the present study, we determined the differential effects of *Wt1* (+KTS) and *Wt1* (−KTS) on ovarian epithelial cell proliferation and migration. The key hub genes and signaling pathways that regulate ovarian epithelial proliferation and migration were explored through transcriptome sequencing and bioinformatics analysis. Then, differential regulatory mechanisms of *Wt1* (+KTS) and *Wt1* (+KTS) were compared, and specific regulatory targets were identified. This study will enrich the screening of early OC biomarkers derived from ovarian epithelial cells and provide new clues for the early diagnosis and precise treatment of OC.

## Results

### Wt1 (+KTS) or Wt1 (−KTS) overexpression vector transduction efficiency and WT1 protein expression analysis

**Fig. 1:**
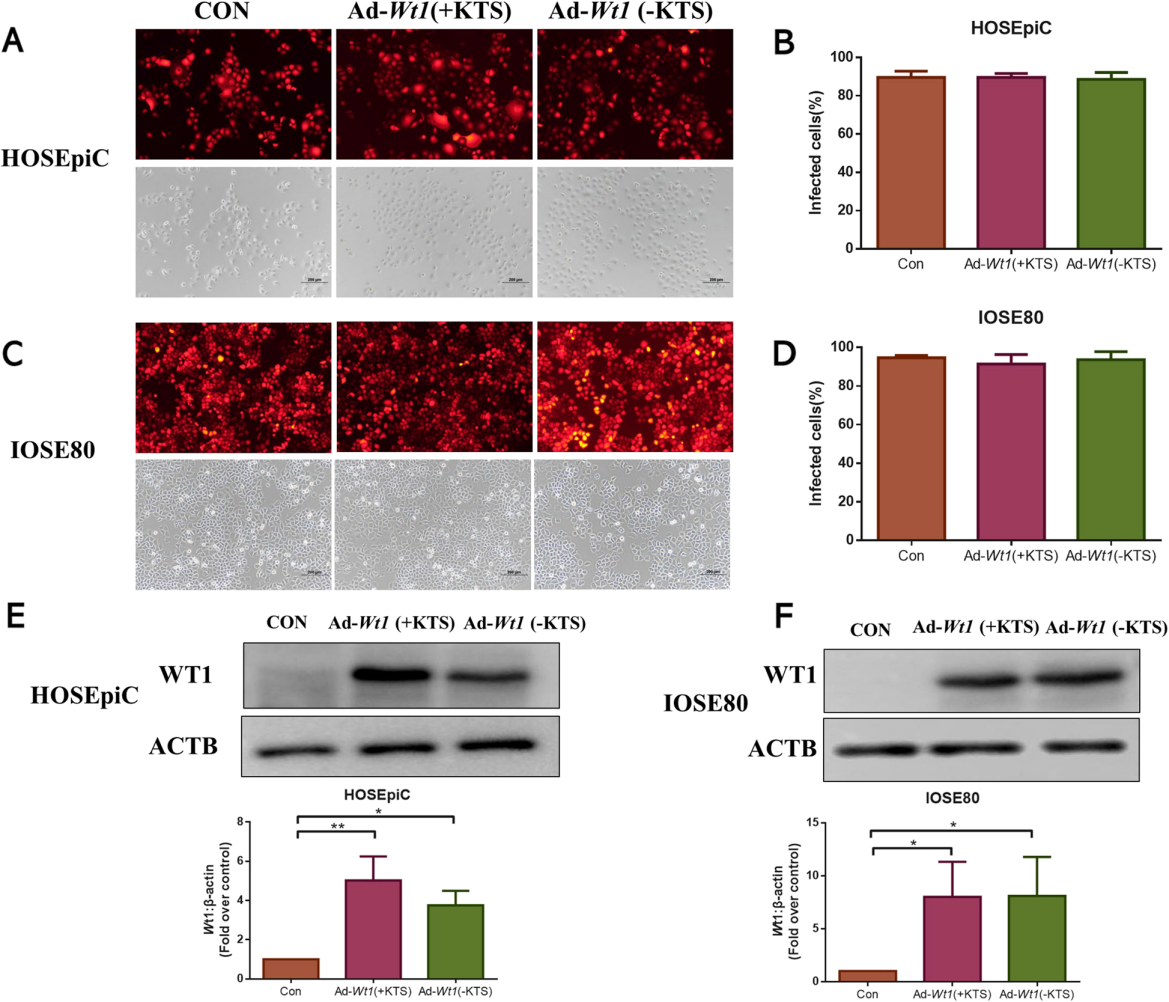
*Wt1* overexpression analysis. Adenovirus containing *Wt1* (+KTS), *Wt1* (−KTS), or the control was used to infect HOSEpiC or IOSE80 cells. **A** Fluorescence images of *Wt1* (+KTS) or *Wt1* (−KTS) overexpression vector in HOSEpiC; **B** Quantitative density analysis of *Wt1* (+KTS) or *Wt1* (−KTS) overexpression in HOSEpiC; **C** Fluorescence images of *Wt1* (+KTS) or *Wt1* (−KTS) overexpression vector in IOSE80; **D** Quantitative density analysis of *Wt1* (+KTS) or *Wt1* (−KTS) overexpression in IOSE80; **E** WB bands and quantitative analysis of WT1 protein expression in HOSEpiC; **F** WB bands and quantitative analysis of WT1 protein expression in IOSE80. *p < 0.05, **p < 0.01. Con: control; Ad: addition

The transduction efficiency of *Wt1* (+KTS) or *Wt1* (−KTS) overexpression vector in HOSEpiC or IOSE80 cells and the effect of WT1 protein expression level were detected by immunofluorescence staining and WB assay. The results are shown in Fig. 1. The transduction efficiency of *Wt1* (+KTS) or *Wt1* (−KTS) overexpression vector in HOSEpiC or IOSE80 cells was > 80% (Fig. 1A-D) and significantly promoted the WT1 protein expression (Fig. 1E, F).

### Overexpression of Wt1 (+KTS) or Wt1 (−KTS) promoted the proliferation and migration of human ovarian epithelial cells, HOSEpiC

**Fig. 2:**
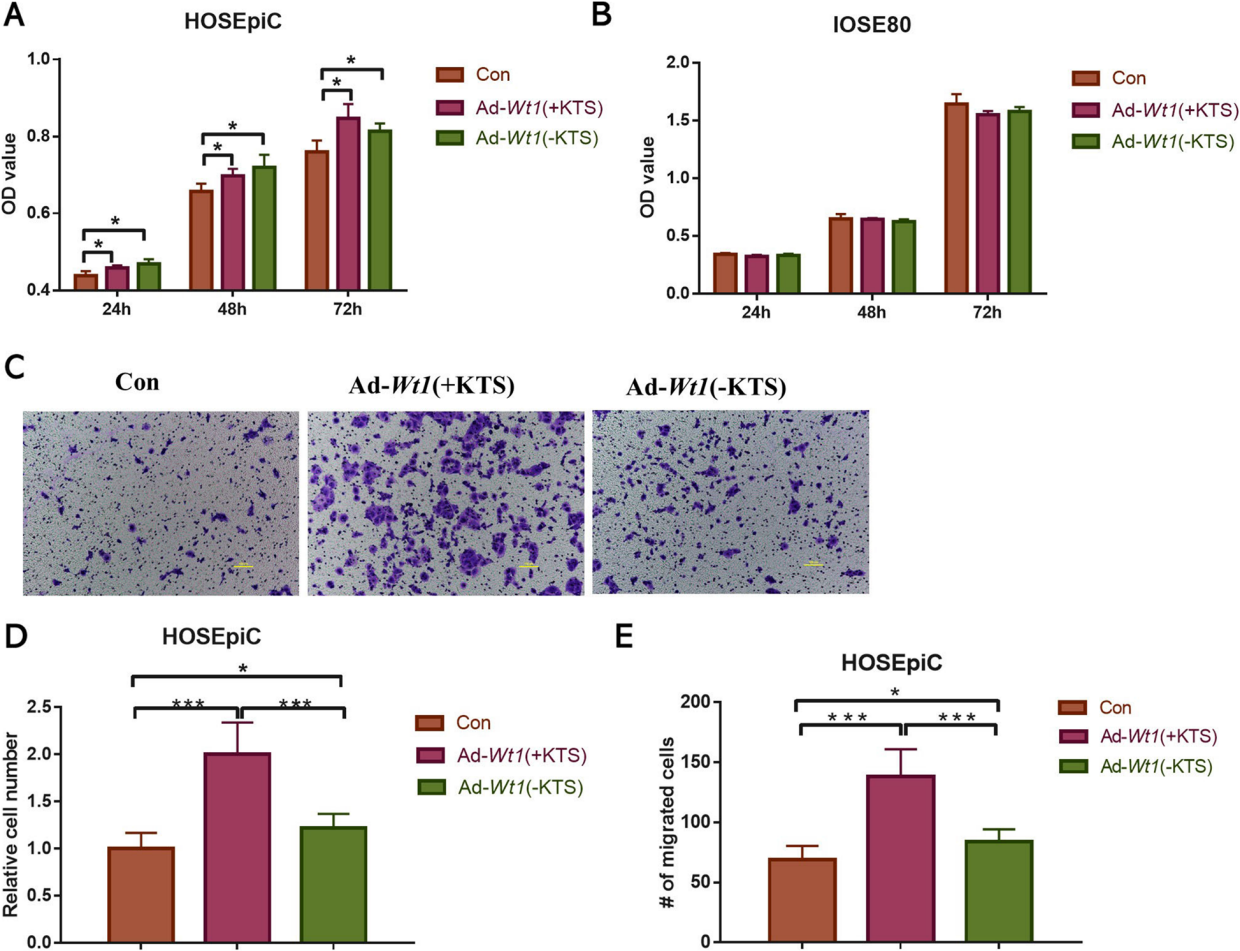
Effects of *Wt1* (+KTS) or *Wt1* (−KTS) on ovarian epithelial cell proliferation and migration. The effects of *Wt1* (+KTS) or *Wt1* (−KTS) overexpression on the proliferation of ovarian epithelial cells were analyzed by CCK-8 and transwell experiments. **A** The effect of upregulation of *Wt1* (+KTS) or *Wt1* (−KTS) on the proliferation of HOSEpiC cells; **B** The effect of upregulation of *Wt1* (+KTS) or *Wt1* (−KTS) on the proliferation of IOSE80 cells; **C** Cell migration images of *Wt1* (+KTS) or *Wt1* (−KTS) overexpression in HOSEpiC cells; **D** Quantitative analysis of cell migration in HOSEpiC cells; **E** Cell number analysis of cell migration in HOSEpiC cells. *p < 0.05, ***p < 0.001. Con: control; Ad: addition

The effects of *Wt1* (+KTS) or *Wt1* (−KTS) overexpression on the proliferation of ovarian epithelial cells (HOSEpiC, IOSE80) were verified by cell counting kit-8 (CCK-8) assay. As shown in Fig. 2A, both *Wt1* (+KTS) and *Wt1* (−KTS) overexpressions significantly promoted the proliferation of HOSEpiC cells. As shown in Fig. 2B, *Wt1* (+KTS) or *Wt1* (−KTS) overexpression had no significant effect on IOSE80 cell proliferation. The effects of *Wt1* (+KTS) or *Wt1* (−KTS) overexpression on HOSEpiC cell migration was verified by transwell experiments. The results are shown in Fig. 2C,D and E, *Wt1* (+KTS) or *Wt1* (−KTS) overexpression significantly promoted HOSEpiC cell migration, and the effects of *Wt1* (+KTS) were more obvious.

### Transcriptome sequencing analysis of HOSEpiC overexpressing Wt1 (+KTS) or Wt1 (−KTS)

The ovarian epithelial cell line HOSEpiC overexpressing *Wt1* (+KTS) or *Wt1* (−KTS) were selected for transcriptome sequencing, and sequencing results were subjected to bioinformatics analysis through the NetworkAnalyst online website. Supplementary Fig. 1 shows the volcano plot of differentially expressed genes (DEGs), and the results showed that the number of DEGs in the *Wt1* (−KTS) group was 400, and the number of DEGs in the *Wt1* (+KTS) group was 252 in HOSEpiC compared with the control group; the number of DEGs in the *Wt1* (−KTS) and *Wt1* (+KTS) groups were 143 and 131, respectively, in IOSE80 compared with the control group.

**Fig. 3:**
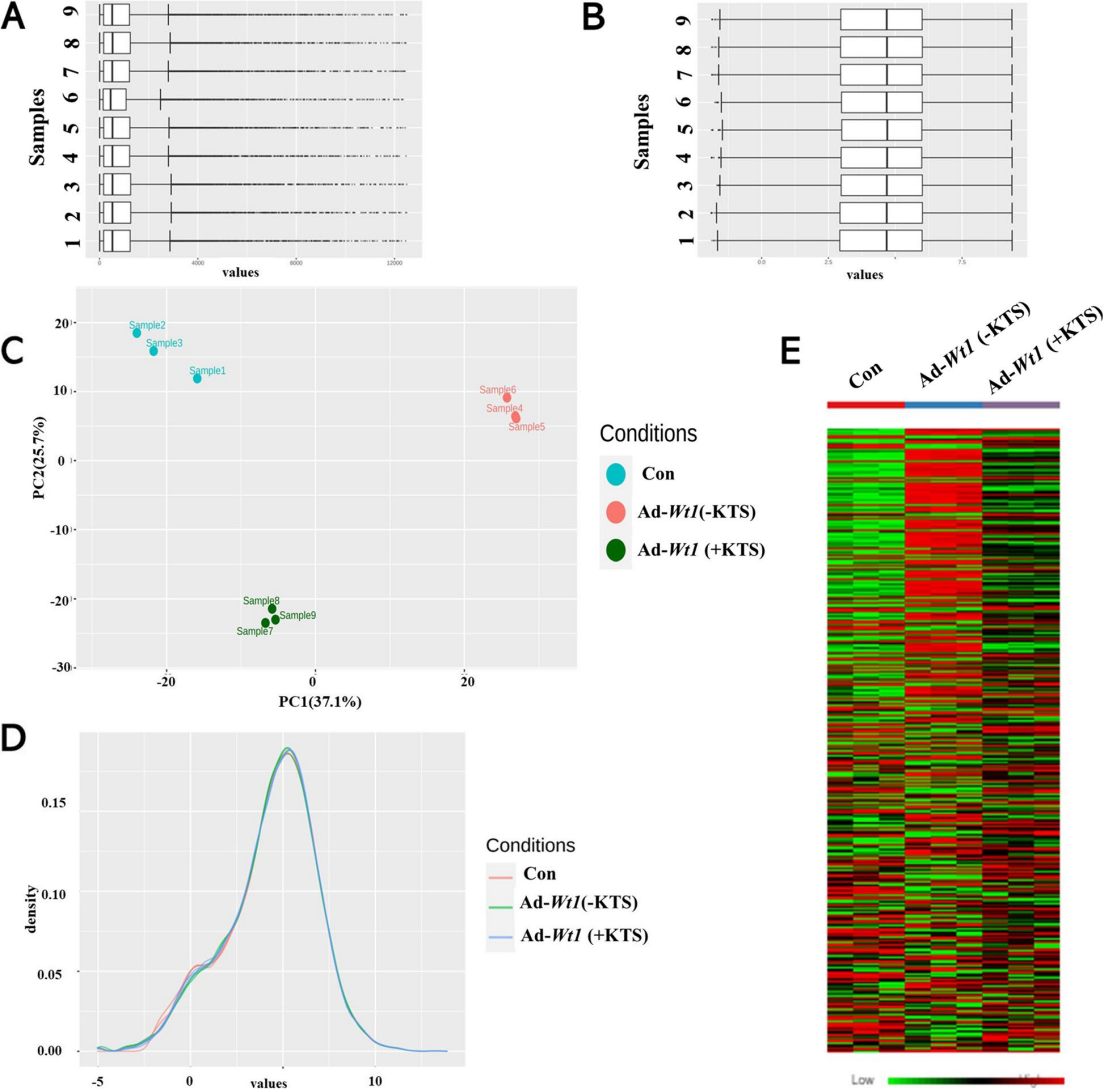
NetworkAnalyst analysis of HOSEpic sequencing data. Transcriptome of HOEpiC cells overexpressing *Wt1*(+KTS) or *Wt1*(−KTS) was sequenced, and the experimental samples were analyzed by bioinformatics through the online website of NetworkAnalyst. **A** Boxplot of counts distribution of each sample before normalization; **B** Boxplot of counts distribution of each sample after normalization; **C** Principal component analysis results; **D** Density distribution of log2counts values in each group; **E** Heat map of *Wt1* (+KTS)/ *Wt1* (−KTS) gene expression. Con: control; Ad: addition

The box plots in Fig. 3A and B showed the distribution of raw read counts. Among them, Fig. 3A shows before normalization, and Fig. 3B shows normalized by Log2-counts per million. The Principal Component Analysis (PCA) plot in Fig. 3C shows the results of the principal component analysis, and the density plot in Fig. 3D shows the density distribution of the log2count values of each group. The analysis results showed that the samples were uniform and reproducible. The heat map in Fig. 3E showed the gene expression profile of *Wt1* (+KTS)/ *Wt1* (−KTS).

### Screening and RT-qPCR validation of genes positively regulating HOSEpiC cell proliferation and migration

**Fig. 4:**
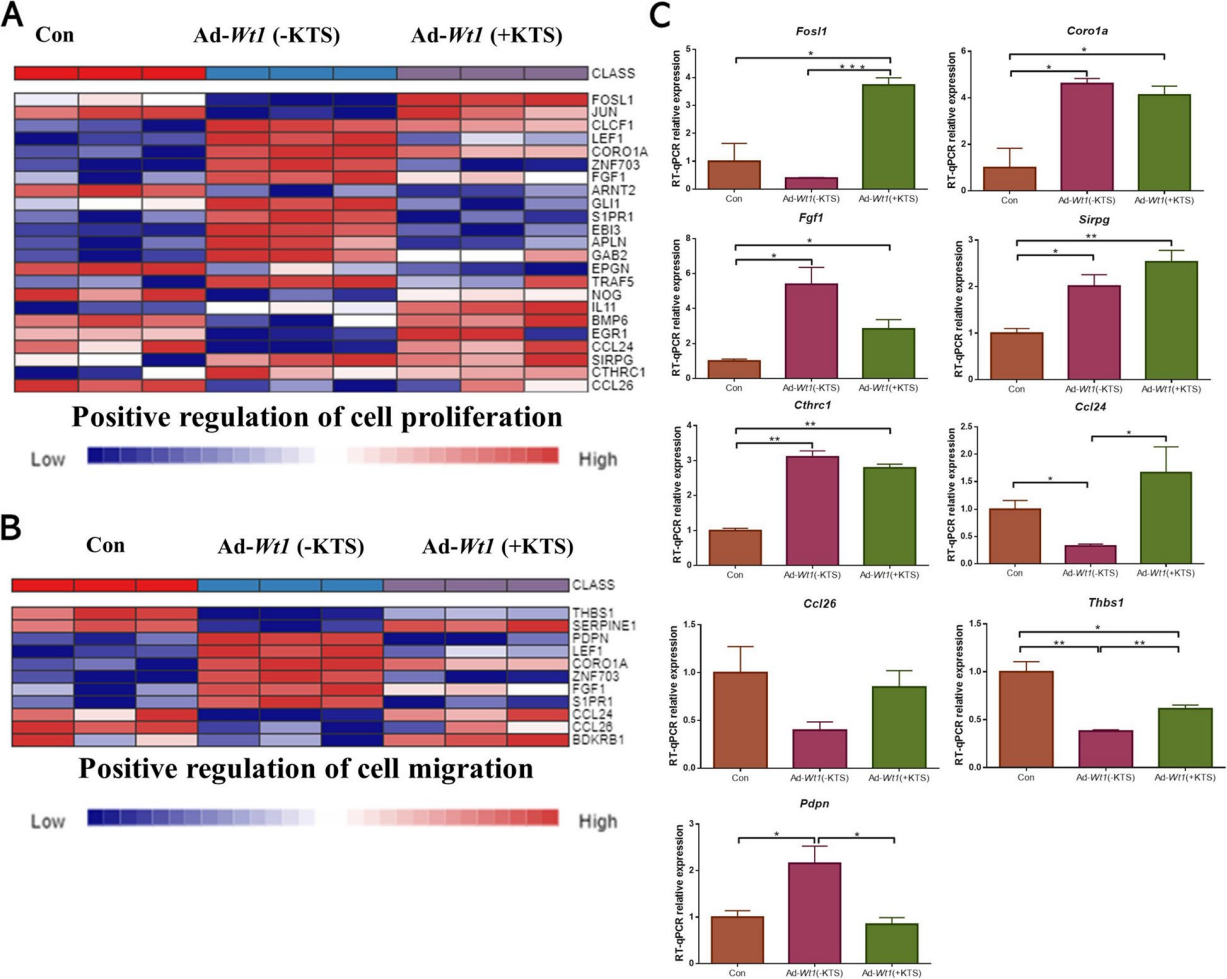
Heatmap and mRNA expression changes of genes positively regulating HOSEpiC cell proliferation and migration. The expression heat map and quantitative verification of genes that positively regulated cell proliferation and cell migration in HOSEpiC cells. **A** The expression heat map of genes enriched in positive regulation of cell proliferation through the NetworkAnalyst online website; **B** The expression heat map of genes enriched in positive regulation of cell migration through the NetworkAnalyst online website; **C** RT-qPCR validation results of nine differentially expressed genes. *p < 0.05, **p < 0.01. Con: control; Ad: addition

Since both *Wt1* (+KTS) and *Wt1* (−KTS) significantly promoted the proliferation and migration of HOSEpiC cells, a heat map was created to display the gene expression enriched in positive-regulated cell proliferation and positive-regulated cell migration (Fig. 4A, B), and DEGs were verified by RT-qPCR (Fig. 4C). These results showed that compared with the control group, both *Wt1* (+KTS)/ *Wt1* (−KTS) had upregulated gene expression, and the number of upregulated genes in *Wt1* (−KTS) was higher. However, in addition to upregulated genes, downregulated genes were observed in *Wt1* (+KTS)/ *Wt1* (−KTS), and the number of downregulated genes was also higher in *Wt1* (−KTS). Thereafter, we selected nine genes such as *Fosl1, Coro1a*, and *Fgf1* for RT-qPCR verification, and the results were consistent with the heatmap results. The above experiments to a certain extent explained the results of our experiments, that is, both *Wt1* (+KTS) and *Wt1* (−KTS) can significantly promote cell proliferation and migration, whereas the effects of *Wt1* (+KTS) were more obvious.

### GO/KEGG analysis of DEGs

**Fig. 5:**
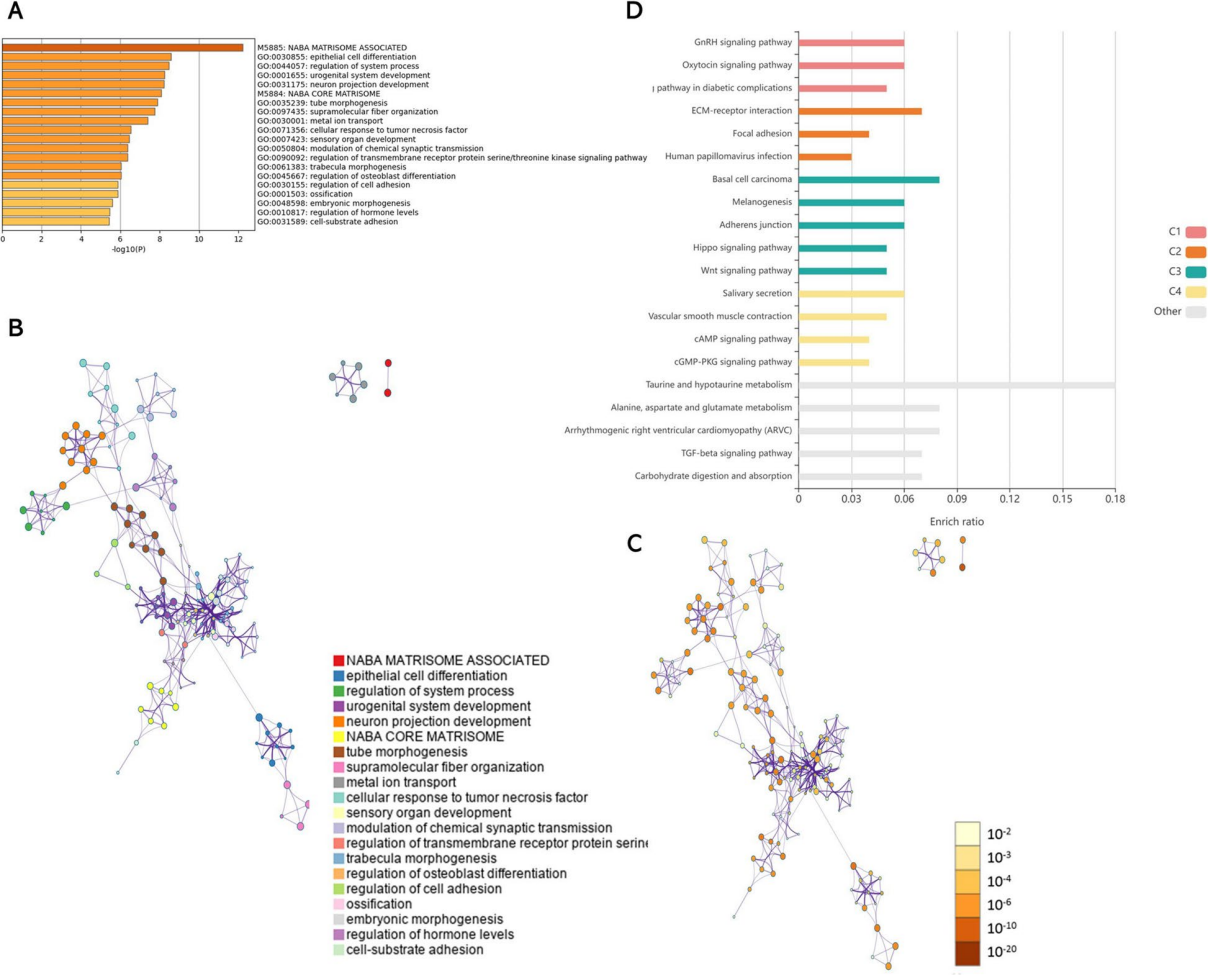
GO/KEGG analysis of differentially expressed genes in *Wt1* (−KTS). Metascape website and KOBAS online software were used to analyze the function and enrichment of differentially expressed genes (DEGs) in *Wt1* (−KTS). **A** Enrichment Analysis of DEGs in *Wt1* (−KTS) by Metascape software. **B** Metascape analysis of network layout. Each term was represented by a circular node with a size proportional to the number of genes, and nodes of the same color belong to the same cluster. **C** Nodes of the same enrichment network were colored by *p*-value. The darker the color, the more statistically significant the node was. **D** KEGG enrichment analysis of DEGs in *Wt1* (−KTS) analyzed by KOBAS software

**Fig. 6:**
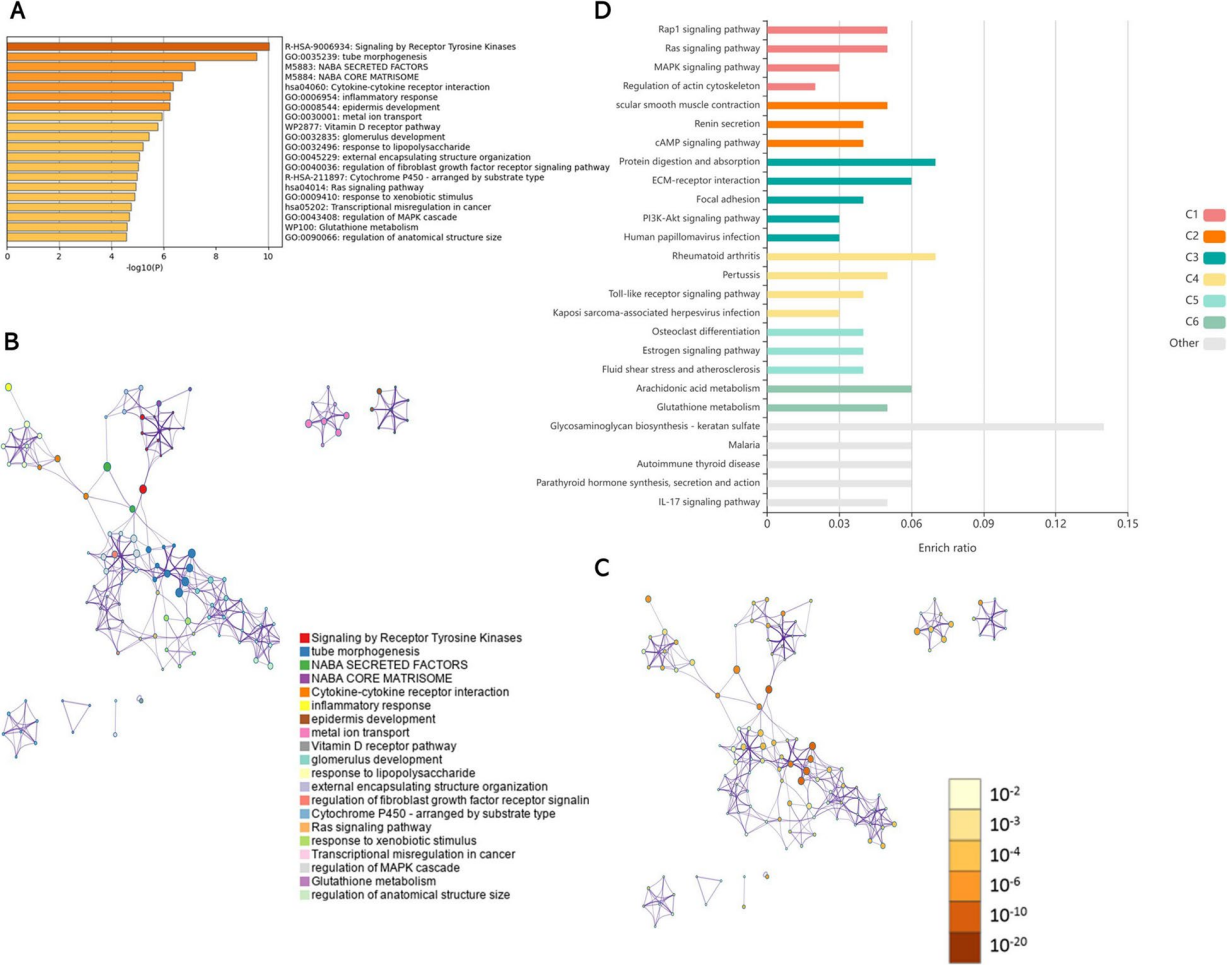
GO/KEGG analysis of differentially expressed genes in *Wt1* (+KTS). Metascape website and KOBAS online software were used to analyze the function and enrichment of differentially expressed genes (DEGs) in *Wt1* (+KTS). **A** Enrichment Analysis of DEGs in *Wt1* (+KTS) by Metascape software. **B** Metascape analysis of network layout. Each term was represented by a circular node with a size proportional to the number of genes, and nodes of the same color belong to the same cluster. **C** Nodes of the same enrichment network were colored by p-value. The darker the color, the more statistically significant the node was. **D** KEGG enrichment analysis of DEGs in *Wt1* (+KTS) analyzed by KOBAS software

We analyzed the DEGs of *Wt1* (+KTS)/ *Wt1* (−KTS) on the Metascape website. The results showed that canonical pathways significantly enriched in *Wt1* (−KTS) DEGs were extracellular matrix (ECM) protein reference datasets (NABA MATRISOME ASSOCIATED, NABA CORE MATRISOME); the biological processes significantly enriched in *Wt1* (−KTS) DEGs were epithelial cell differentiation, system process regulation, urogenital system development, neuron projection development, tube morphogenesis, etc. (Fig. 5A–C). The Reactome Gene Sets, which significantly enriched *Wt1* (+KTS) DEGs, were Signaling by Receptor Tyrosine Kinases; the biological processes that significantly enriched in *Wt1* (+KTS) DEGs were tube morphogenesis, inflammatory response, epidermis development, metal ion transport, etc.; the canonical pathway that significantly enriched in DEG pathways was a reference dataset of ECM proteins (NABA SECRETED FACTORS, NABA CORE MATRISOME); the KEGG pathway where DEGs were significantly enriched was cytokine–cytokine receptor interaction and Ras signaling pathway (Fig. 6A–C).

Next, KEGG enrichment analysis on DEGs of *Wt1* (+KTS)/ *Wt1* (−KTS) was performed using KOBAS online software. The results showed that among the KEGG pathways significantly enriched in DEGs of *Wt1* (−KTS), the top cluster associated with proliferation and migration was cluster 2, mainly the ECM–receptor interaction and focal adhesion (Fig. 5D). Among the KEGG pathways significantly enriched in DEGs of *Wt1* (+KTS), the top cluster associated with proliferation and migration was cluster 1, mainly Ras, Rap1, and MAPK signaling pathways (Fig. 6D).

### Screening and expression verification of the hub gene

**Fig. 7:**
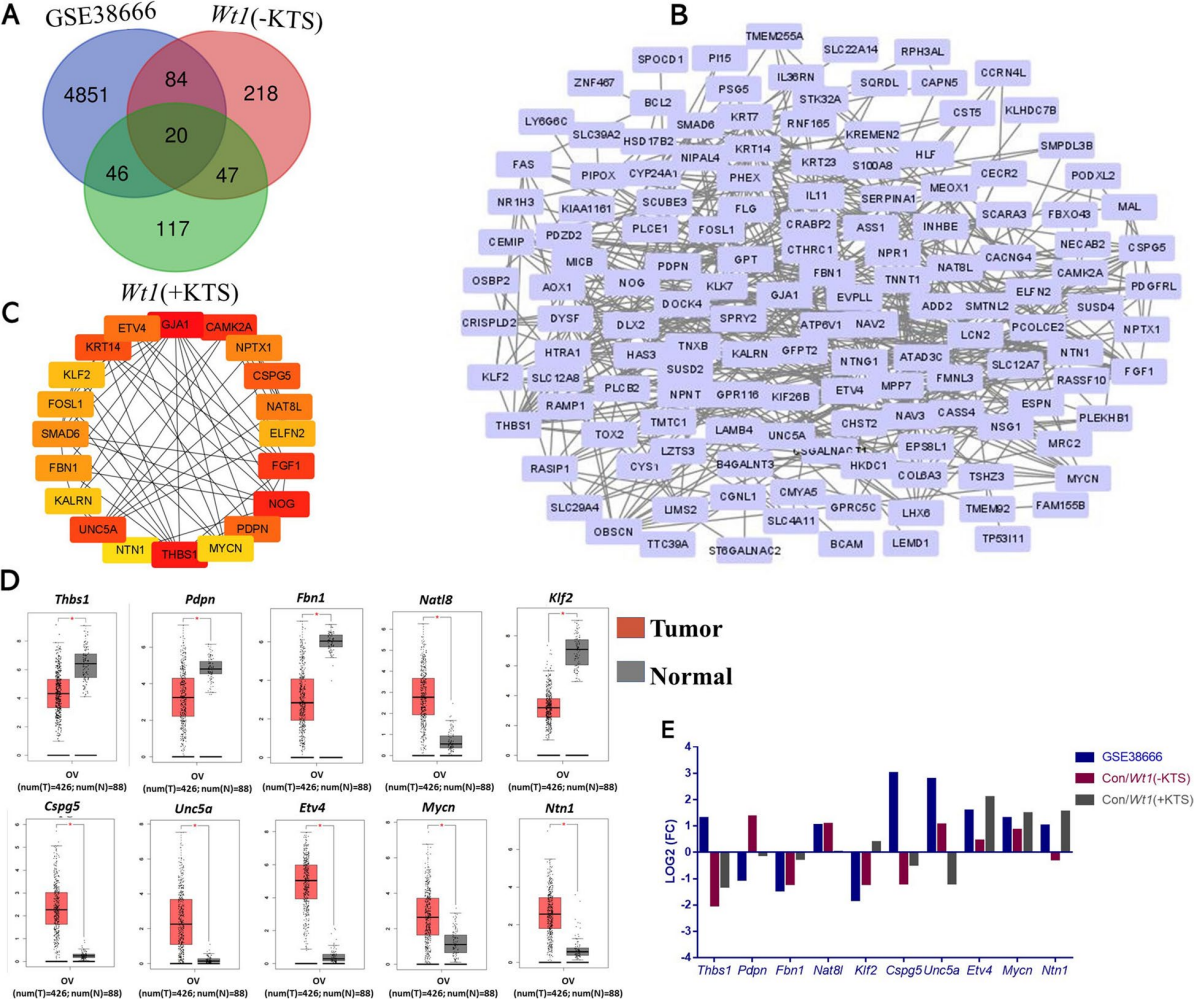
Screening and expression of PPI network and hub gene. Combining the DEGs between normal ovarian surface epithelium and OCE samples in GEO dataset GSE38666, the intersection genes of GSE38666 and *Wt1*(+KTS) and the intersection genes of GSE3866 and *Wt1* (+KTS) were screened. 150 DEGs were selected for PPI network analysis and hub gene screening. **A** Venn analysis plot of DEGs inthree datasets; **B** PPI regulatory network of 150 intersecting genes; **C** The hub gene regulatory network of top20; **D** Ten hub genes that were significantly differentially expressed between ovarian cancer tissues and normal tissues; **E** The expression of the above 10 genes in the three sets of sequencing data

As shown in Fig. 7A, a total of 150 intersection genes were observed between the DEGs of variant cancer epithelium (OCE) and normal tissue samples in GSE38666 and DEGs of *Wt1* (−KTS)/ *Wt1* (+KTS). The protein–protein interaction (PPI) regulatory network in Fig. 7B showed complex interactions among the 150 intersecting genes. Figure 7C shows the top 20 hub genes calculated by the cytoHubba plugin in Cytoscape. GEPIA online software analysis showed that *Thbs1*, *Pdpn*, *Fbn1*, *Nat8l*, *Klf2*, *Cspg5*, *Etv4*, *Unc5a*, *Mycn*, and *Ntn1* were significantly differentially expressed between OC and normal tissues (Fig. 7D). The results in Fig. 7E showed that except for the genes with the same expression trend, *Pdpn* and *Cspg5* expressions in *Wt1* (−KTS) were inconsistent with the results of GEPIA analysis and GSE38666 sequencing; the *Unc5a* expression in *Wt1* (+KTS) was opposite to the results of GEPIA analysis and GSE38666 sequencing.

### The hub gene expression was related to the prognosis and clinicopathological features of OC

**Fig. 8:**
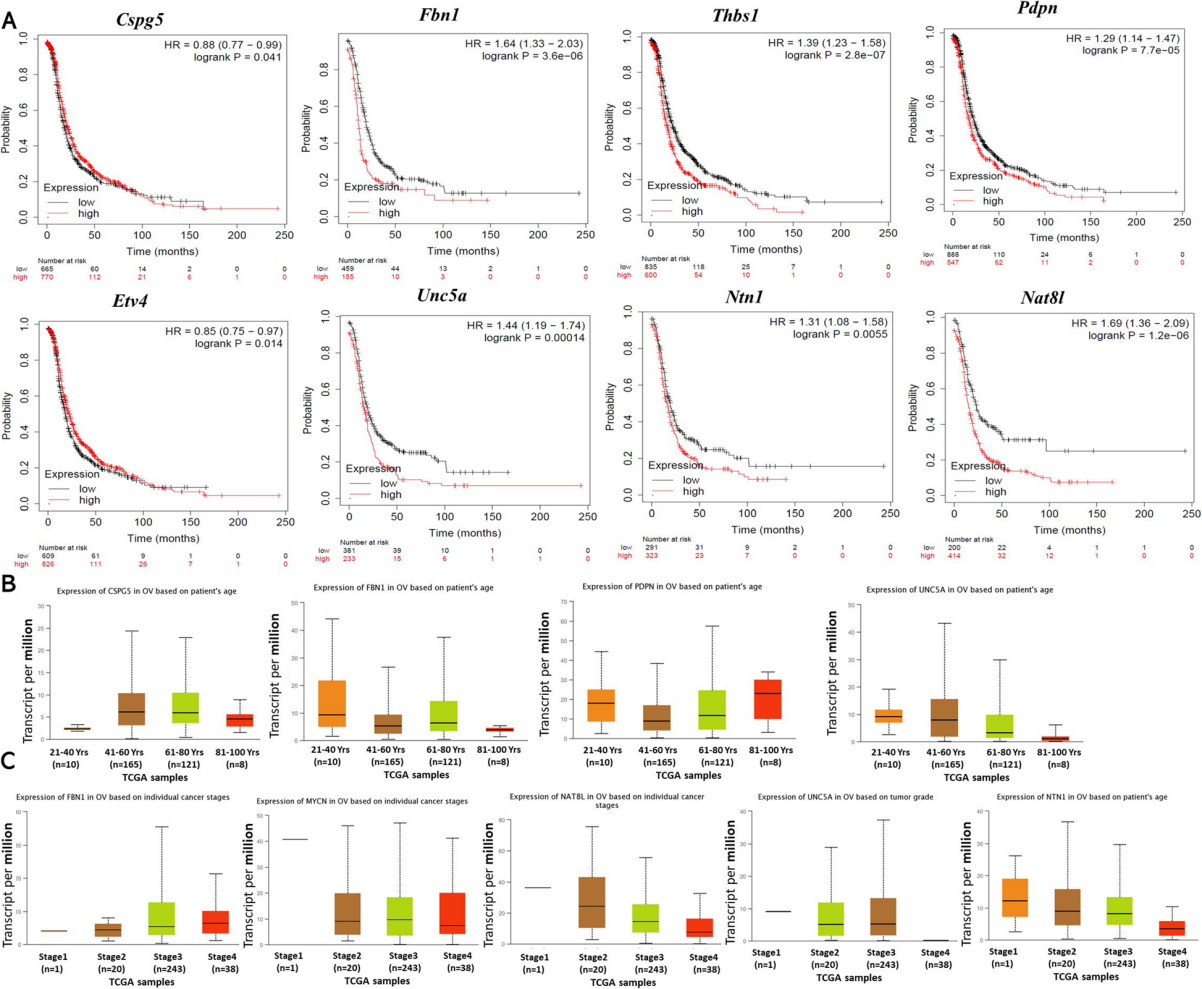
Prognosis and clinicopathological features of hub gene in ovarian cancer. Kaplan-Meier mapper and UALCAN online website were used to analyze the prognosis and clinical pathological features of hub genes. **A** Hub genes significantly associated with ovarian cancer prognosis; **B** Hub genes significantly correlated with age in ovarian stage patients; **C** Hub genes significantly associated with individual cancer stage in ovarian stage patients

The results in Fig. 8A showed that *Cspg5*, *Fbn1*, *Thbs1*, *Pdpn*, *Etv4*, *Unc5a*, *Ntn1*, and *Nat8l* expressions were significantly correlated with the prognosis of patients with OC. The results in Fig. 8B showed that *Cspg5*, *Fbn1*, *Pdpn*, and *Unc5a* expressions were significantly correlated with the age of patients with OC. The results in Fig. 8C showed that the *Fbn1*, *Mycn*, *Nat8l*, *Unc5a*, and *Ntn1* expressions were significantly correlated with the individual cancer stage of OC.

## Discussion

In this study, the role of the *Wt1* gene in the development of ovarian epithelium to OC was selected for two ovarian epithelial cell lines, HOSEpiC and IOSE80, to evaluate the effects of *Wt1* on ovarian epithelial cell proliferation and migration. Our study showed that *Wt1* promoted the process of HOSEpiC proliferation and migration; however, no significant effect was observed on IOSE80 proliferation. In this regard, we performed transcriptome sequencing of both cell lines, and in HOSEpiC, *Wt1* (+KTS) significantly altered the expression of 252 genes and *Wt1* (−KTS) significantly altered the expression of 400 genes, whereas, in IOSE80 among them, *Wt1* (+KTS) significantly altered the expression of 131 genes and *Wt1* (−KTS) significantly altered the expression of 143 genes. Compared with HOSEpiC, *Wt1* affected the IOSE80 gene expression in a smaller number and had no significant effect on cell proliferation. This difference may be associated with different properties of the two types of ovarian epithelial cells. IOSE80 cells expressing SV40 large T antigen, which can inactivate the p53 and pRb pathways, make IOSE80 immortalized nontumorigenic ovarian surface epithelial cell lines [24–26], and HOSEpiC are human normal ovarian epithelial cells [27–29]. We also found that the proliferation rate of IOSE80 was significantly higher than that of HOSEpiC during the culture process, which may be caused by SV40 large T antigen that allowed the IOSE80 cell line to escape the growth control and proliferate too fast [30]. Therefore, compared with IOSE80, HOSEpiC can better represent the real situation of ovarian epithelial cells. During the follow-up, we mainly take HOSEpiC as the research object to determine the specific role of *Wt1* in ovarian epithelial cells.

Studies have shown that the *Wt1* gene played an important role in normal genitourinary development and cancer development, and approximately 10% of Wilms’ tumors showed *Wt1* mutations [31]. Wei L et al. found that *Wt1* overexpression plays a role in the proliferation and migration of glioblastoma multiforme [32]. Overexpression of *Wt1*-AS inhibited the proliferation and migration of gastric and cervical cancer cells [33, 34]. However, studies had also shown that in pancreatic and OCs, *Wt1* did not play a significant role in proliferation [16, 35]. In our study, we found that *Wt1* promoted the proliferation and migration of ovarian epithelial cells HOSEpiC, indicating that *Wt1* may play an important role in the transformation of ovarian epithelium to OC.

We further investigated the specific roles of *Wt1* (+KTS) and *Wt1* (−KTS) spliced isoforms of the *Wt1* gene in the proliferation and migration of ovarian epithelial cells, and the results showed that *Wt1* (+KTS) and *Wt1* (−KTS) both promote the proliferation and migration of HOSEpiC. However, *Wt1* (+KTS) surprisingly promoted HOSEpiC migration more strongly than *Wt1* (−KTS). Conversely, our previous studies in follicular granulosa and theca cells showed that *Wt1* (−KTS) plays a stronger role in regulating progesterone secretion [22, 23]. In leukemia (acute myelocytic leukemia), *Wt1* (+KTS) can promote cancer cell proliferation, whereas *Wt1* (−KTS) inhibited cell growth and proliferation and promoted cell apoptosis due to the expression of genes encoding the apoptosis regulator Caspase 9 [36, 37]. The reasons for different results may be related to differences in the cell characteristics in different tissues. Furthermore, the *Wt1* function was bidirectional. *Wt1* can act as both a tumor suppressor gene and a proto-oncogene, which also included various splice isoforms, the function was more complex, which may also be the reason for the above results. This also showed that it was of great significance to examine the functions of *Wt1* and its spliced isoforms in different diseases.

To further understand the specific mechanisms of *Wt1* (+KTS) and *Wt1* (−KTS) in ovarian epithelial cell proliferation and migration, transcriptome sequencing analysis was performed on HOSEpiC. Compared with the control group, *Wt1* (−KTS) affected DEGs more than *Wt1* (+KTS). Since both *Wt1* (+KTS) and *Wt1* (−KTS) significantly promoted the proliferation and migration of epithelial cells, a heatmap display of gene expression enriched in positive regulation of cell proliferation and positive regulation of cell migration was performed, and their differences were analyzed. Expressed genes were quantitatively validated. Our results suggested that the cell proliferation promotion by *Wt1* (+KTS) may be associated with the upregulation of positively regulated proliferation-related genes *Fosl1*, *Coro1a*, *Fgf1*, *Sirpg*, *Cthrc1*, and *Ccl24*; *Wt1* (−KTS) had a more obvious upregulation effect on *Coro1a*,*Fgf1*, *Cthrc1*, and other positively regulated proliferation-related genes; however, the downregulation of *Fosl1*, *Ccl24*, *Ccl26,* and other positively regulated proliferation-related genes resulted in a lower promotion effect on ovarian epithelial cells than *Wt1* (+KTS). In terms of cell migration, *Wt1* (+KTS) promoted the *Coro1a*, *Ccl24*, and *Fgf1* expressions, the key genes that positively regulate cell migration and inhibited the *Thbs1* expression, whereas *Wt1* (−KTS) promoted the *Coro1a*, *Fgf1*, and *Pdpn* expressions but also inhibited the expression of more key genes that positively regulated cell migration, such as *Thbs1*, *Ccl24*, and *Ccl26*, among others. This also explained the results of our experiment to a certain extent, that is, both *Wt1* (+KTS) and *Wt1* (−KTS) significantly promoted the cell migration process; however, the effects of *Wt1* (+KTS) were more obvious.

To further understand the functional differences between *Wt1* (−KTS) and *Wt1* (+KTS) in regulating ovarian epithelial cell proliferation and migration, KEGG enrichment analysis was performed on DEGs of *Wt1* (−KTS)/ *Wt1* (+KTS) by KOBAS online software, and DEGs of *Wt1* (−KTS) were significantly enriched in ECM–receptor interaction and focal adhesion. *Wt1* (−KTS) may affect cell proliferation and migration through these two pathways. ECM can mediate the expression of focal adhesion-related genes, and their expression products can bind to cell surface receptors with ECM and transmit extracellular signals to the cell, thereby affecting cell transfer [38]. Studies have shown that in colorectal cancer (CRC) cells, miR-215-5p inhibited the metastatic ability of CRC cells by regulating the ECM–receptor interaction and focal adhesion pathway, thereby reducing the CRC incidence [39]; simultaneously, some studies had shown that esophageal squamous cell carcinoma mutant genes were mainly enriched in the ECM–receptor interaction and focal adhesion signaling pathways, this signaling pathway was also enriched in multiple myeloma, which controlled the cancer cell metastasis by affecting cell adhesion [40, 41]. LIM domain 2 (LIMD2) also affected the proliferation and metastasis of OC cells by regulating their focal adhesion pathway [42].

The DEGs of *Wt1* (+KTS) were significantly enriched in Rap1, Ras, and MAPK signaling pathways. Previous studies demonstrated that SHARPIN, which was highly expressed in melanoma tissues, could mediate the activity of its downstream pathways (p38, JNK/c- Jun), thereby promoting the proliferation and metastasis of cancer cells [43]. Simultaneously, the Rap1 signaling pathway played an important mediating role in non-small cell lung cancer, nasopharyngeal carcinoma, and chronic lymphocytic leukemia, affecting the metastatic process of cancer cells [44–46]. In OC, the MAPK signaling pathway played an important role. Perez-Juarez CE et al. found that in ovarian clear cell carcinoma tumor cells, the expression of precursor granulin was regulated by the ERK1/2 signaling pathway in the MPAK pathway, which in turn affected the cancer cell proliferation [47]. Yang Liu et al. found that overexpressed epithelial membrane protein 1 in EOC could activate the MAPK pathway, thereby promoting the proliferation and metastasis of OC cells and could further enhance the EMT occurrence [48]. Prostaglandin E2 receptor EP3 in OC cells also activated this signaling pathway to promote cancer cell proliferation [49]. In other cancers, Ras-MAPK was also involved in cancer cell proliferation and metastasis. Relevant studies reported that the overexpression of long non-coding RNA H19 could further activate the Ras-MAPK signaling pathway by upregulating the Ras activity and promoting the migration and invasion of CRC cancer cells [50]. Furthermore, studies had found that Asparanin A further inhibited the function of the Ras-MPAK pathway by affecting the miRNA expression in cells, thereby inhibiting the EC metastasis of endometrial cancer cells [51]. In HCC cells, heterogeneous nuclear ribonucleoprotein C inhibited the epithelial-mesenchymal transition through the Ras/MAPK signaling pathway, and arrested the cells in the G0/G1 phase, thereby inhibiting the cancer cell metastasis and proliferation [52]. Meanwhile, PD-1 activation in thyroid cancer cells could also activate the Ras-MAPK signaling pathway, thereby promoting cancer cell proliferation [53]. The above analysis indicated that functional differences between *Wt1* (−KTS) and *Wt1* (+KTS) in regulating ovarian epithelial cell proliferation and migration may be due to differences in their DEGs and significantly enriched signaling pathways, and its specific regulation mechanism should be investigated further.

To screen the key regulatory genes that may regulate the malignant transformation of ovarian epithelial cells by *Wt1* (−KTS)/ *Wt1* (+KTS), we performed Venn analysis on DEGs of the OC-related sequencing dataset GSE38666 and DEGs of this study and further screened out 10 DEGs between OC and normal tissues through the PPI network construction and hub gene screening. Among them, *Cspg5*, *Fbn1*, *Thbs1*, *Pdpn*, *Etv4*, *Unc5a*, *Ntn1*, and *Nat8l* were associated with the OC prognosis; *Cspg5*, *Fbn1*, *Pdpn*, and *Unc5a* were correlated with patient age; *Fbn1*, *Mycn*, *Nat8l*, *Unc5a*, and *Ntn1* were significantly correlated with individual cancer stage. Furthermore, the expression of *Pdpn* and *Cspg5* in *Wt1* (−KTS) was inconsistent with the results of GEPIA analysis and GSE38666 sequencing, the *Unc5a* expression in *Wt1* (+KTS) was inconsistent with the results of GEPIA analysis and GSE38666 sequencing. *Cspg5* is a proteoglycan with an EGF-like module that acts as a neuronal growth and differentiation factor, and a recent study showed that *Cspg5* could be used to predict the EOC prognosis [54]. *Pdpn* was expressed in various types of human tumors, and some reports suggested that *Pdpn* can promote tumor cell migration, invasion, and metastasis, contributing to cancer progression [55, 56]. Moreover, studies revealed that *Unc5a* is associated with many types of cancer and acted as a tumor suppressor in non-small cell lung cancer [57], may serve as a new early marker in prostate cancer [58], and restricted the metastasis of breast cancer cells [59]. We, therefore, hypothesized that these 10 hub genes may be the key regulatory genes for *Wt1* (−KTS)/ *Wt1* (+KTS) to regulate the malignant transformation of ovarian epithelial cells, whereas *Pdpn*, *Cspg5*, and *Unc5a* may serve as *Wt1* (+/−KTS) variant key genes for functional differences, differential diagnosis, and specific clinical targets.

In conclusion, we studied the effect of *Wt1* (+/−KTS) on the proliferation and migration of ovarian epithelial cells, analyzed the differential regulation of two splicing isoforms, and screened out the hub genes and key signal pathways that may regulate the malignant transformation of ovarian epithelial cells. This study explored the regulation of *Wt1* (+/−KTS) at the gene level, which has important guiding significance for revealing the pathogenesis of OC from the source. We will further study the specific molecular mechanism of *Wt1* (+/−KTS) and related hub genes in the occurrence and development of OC, to further promote the early diagnosis and the development of new therapeutic targets of OC.

## Materials and methods

### Cell culture

Human ovarian epithelial cells include cell lines such as HOSEpic, HOSE-6-3, HOSE11-12, IOSE80, IOSE29, IOSE121 and IOSE364 [27, 60–62], of which HOSEpiC and IOSE80 are two cell lines that are easy to culture and frequently used. Human normal ovarian epithelial cells (HOSEpiC) and ovarian surface epithelial cells expressing SV40 large T antigen (IOSE80) were purchased from BeNa Culture Collection. The cells were cultured in RPMI-1640 medium (Hyclone) supplemented with 10% fetal bovine serum (FBS) (GIBCO) and 1% antibiotic-antimycotic solution. Cells were seeded either in 96-well plates for the proliferation experiment, 24-well plates for immunofluorescence and cell migration assay, or 35-cm cell culture dishes for mRNA expression and protein expression analysis. The cells were used in the experiment after normal passage twice. The mRNA and protein extracts were stored in a refrigerator at − 80 °C for use. Cell culture was performed with at least triplicate wells of the cells, and the experiment was repeated three times.

### Wt1 (+/−KTS) overexpression

*Wt1* (+/−KTS) overexpression was performed as in our previous study with minor modifications [23]. Briefly, *Wt1* (+KTS) (NM_024426) and *Wt1* (−KTS) (NM_024424) sequences were provided to Hanbio Biotechnology. The genes were inserted into pAdEasy-EF1-MCS-3flag-CMV-mCherry, and then high titers (10^10^PFU/ml) of adenovirus were obtained (Hanbio Biotechnology, Shanghai, China). Adenovirus containing *Wt1* (+KTS), *Wt1* (−KTS), or the control was used to infect ovarian epithelial cells based on the viral infection procedure of Hanbio Biotechnology.

### RNA isolation and cDNA preparation

After adding Trizol to the cells and letting them stand for 5 min at room temperature, it was extracted into a centrifuge tube. The total RNA was extracted by adding chloroform, isopropanol, and absolute ethanol, respectively, and centrifuged at 4 °C. To purify the obtained RNA, 75% ethanol was added for centrifugation and washed twice. Finally, the RNA was resuspended in DEPC water, and the RNA concentration was measured. We used the PrimeScript™ RT reagent kit with gDNA Eraser (Takara) kit to reverse-transcribe the extracted RNA to synthesize cDNA. Samples were stored in a − 20 °C freezer.

### Real-time quantitative polymerase chain reaction

Real-time quantitative polymerase chain reaction (RT-qPCR) was used to detect the expression of proliferation and migration-related genes and validate the sequencing data. The specific primers and TB GreenTM Premix Ex Taq TM (TaKaRa) were used for RT-qPCR. Actin B (*Actb*) was used as the internal control. The comparative CT method was used to calculate the relative quantity of the mRNAs and the 2 − ΔΔCT was used to calculate the fold change. The selected pairs of primers are shown in Table 1. Specific procedures used for RT-qPCR were 95 °C for 30 s; followed by 95 °C, 5 s; 57 °C, 30 s; and 72 °C, 30 s. Melting curves were run at 60 °C, with a temperature increase of 0.5 °C every 5 s until the cycle was stopped at 95 °C.

**Table 1:** Sequences for gene primers

Gene Symbol	RT Forward Primer(5′- 3′)	RT Reverse Primer(5′- 3′)	Accession Number	Product Length (bp)
*Fosl1*	CAGGCGGAGACTGACAAACTG	TCCTTCCGGGATTTTGCAGAT	NM_005438.5	132
*Coro1a*	CTGTGCTGTCAACCCTAAGTTT	GTGGGCGCATTCTTGTCCA	NM_001193333.3	111
*Fgf1*	GGCTCACAGACACCAAATGA	GGCCAACAAACCAATTCTTCTC	NM_000800.5	109
*Sirpg*	CCTTTCCTGCTTCTGACTCTAC	GAGTGGCTGTCTTTCCAACT	NM_018556.4	115
*Cthrc1*	CAATGGCATTCCGGGTACAC	GTACACTCCGCAATTTTCCCAA	NM_138455.4	168
*Ccl24*	ACATCATCCCTACGGGCTCT	CTTGGGGTCGCCACAGAAC	NM_001371193.1	176
*Ccl26*	AGACCTGCTGCTTCCAATAC	GGTACAGACTTTCTTGCCTCTT	NM_001371936.1	131
*Thbs1*	AGACTCCGCATCGCAAAGG	TCACCACGTTGTTGTCAAGGG	NM_003246.4	157
*Pdpn*	GTGTAACAGGCATTCGCATCG	TGTGGCGCTTGGACTTTGT	NM_006474.5	80
*Actb*	CTGGAACGGTGAAGGTGACA	AAGGGACTTCCTGTAACAACGCA	NM_001101.5	140
*Gadph*	AAGGTGAAGGTCGGAGTCAAC	GAAGGGGTCATTGATGGCAAC	NM_002046.7	105

### Western blotting

Western blotting was performed as in previous studies with minor modifications [63, 64]. Briefly, cells were lysed on ice for 20 min using a mixture containing RNA lysis buffer and phosphatase inhibitor. Thereafter, 5 × SDS was added and boiled for 10 min in a 100 °C metal water bath. Equal amounts of samples were added to 10% sodium dodecyl sulfate-polyacrylamide gel electrophoresis to separate proteins and then transferred to polyvinylidene fluoride membranes. After applying the blocking solution (Beyotime-QuickBlock Western Blocking Solution), the membrane and rabbit anti-Wilms’ tumor protein (1:1000; Cell Signaling Technology, 83,535) were incubated overnight at 4 °C. Following three washes with tris-buffered saline, the membrane was incubated with a secondary antibody (abclonal murine monoclonal antibody) for 1 h at room temperature. Finally, the luminescent solution (Millipore, WBKLS0100) was used to expose the target band protein.

### Proliferation experiments

Proliferation experiments was performed as in previous studies with minor modifications [16]. Briefly, after counting the cells with a cell counting plate, the cells were seeded in a 96-well plate at the number of 2 × 103/well and were subjected to overexpression treatment after 24 h. We used Cell Counting Kit-8 (CCK-8) for cell proliferation experiment. Proliferation experiments were performed at 24 h, 48 h, and 72 h, respectively. The details were that the culture solution containing CCK solution (1:10) was added to the 96-well plate, incubated in the incubator for 1 h, and finally, a 450 nm absorbance value was detected with a microplate reader.

### Migration experiment

Proliferation experiments was performed as in previous studies with minor modifications [16]. Briefly, the transwell chambers were placed in a clean 24-well plate, and the cells transduced for 24 h were seeded into the upper chamber of the transduction well at a density of 5 × 10^4^ cells/well. The upper chambers were serum-free culture medium, whereas the lower chambers were added with a 10% FBS culture medium. After culturing in a 37 °C, 5% CO_2_ incubator for 24 h, cells were fixed with 4% paraformaldehyde, stained with 0.1% crystal violet, and the results of the microscope photographs were analyzed with the ImageJ software.

### RNA sequencing

The sequencing platform of this study was DNBSEQTM, and the sequencing length was PE150. The raw data of sequencing contained reads with low quality, adapter contamination, and high N content of unknown bases. These reads should be removed before data analysis to ensure the reliability of results. This project used the filtering software SOAPnuke independently developed by BGI for filtering. In this study, a total of nine samples were measured using the DNBSEQ platform, and each sample produced an average of 6.54 G of data. The average alignment rate of the sample alignment genome and aligned gene set was 92.01 and 77.48%, respectively. Species name: *Homo sapiens*, source: NCBI, reference genome version: GCF_000001405.39_GRCh38.p13.

### NetworkAnalyst

The sequencing data of HOSEpiC and IOSE80 cells were analyzed via the NetworkAnalyst online website (https://www.networkanalyst.ca/) [65]. Through the variance filter, the genes with a variance percentage of < 15 were filtered out, whereas, through the low abundance filter, the genes with count values of < 4 were filtered out. Log2-counts per million were selected as the normalization method. Differential expression analysis was performed using the statistical method of EdgeR. We set the adjusted *p*-value of < 0.05 and |log2-fold change| > 1 to identify DEGs. We analyzed the gene expression of HOSEpic and IOSE80 using the volcano plot. The gene expression of HOSEpic was further analyzed by the heatmap, and the genes that positively regulate cell proliferation and migration were screened by enrichment analysis.

### GO/KEGG analysis

The DEGs of *Wt1* (+KTS) or *Wt1* (+KTS) of HOSEpiC obtained by NetworkAnalyst analysis were analyzed using the Metascape online software (https://metascape.org/), and the top 20 clusters were selected for display [66]. The above genes were subjected to the KEGG pathway enrichment analysis by KOBAS online software (http://kobas.cbi.pku.edu.cn/), and the corrected *p*-value of < 0.05 was significantly enriched [67].

### Hub genetic screening

To compare the expression differences between *Wt1* (+KTS) and *Wt1* (+KTS) overexpressions in ovarian epithelial cells and EOC tissues, we selected a GEO dataset GSE38666, in which the samples of normal ovarian surface epithelium and OCE were selected for analysis and comparison. Sequencing data were analyzed, and DEGs were screened through the NetworkAnalyst online website. We used the Draw Venn Diagram online website (http://bioinformatics.psb.ugent.be/webtools/Venn/) to perform Venn analysis on the differential gene data in the above three datasets, respectively. A total of 150 DEGs including intersection genes of GSE38666 and *Wt1* (+KTS) and intersection genes of GSE38666 and *Wt1* (+KTS) were selected for subsequent analysis. The PPI regulatory network of 150 DEGs was constructed through the STRING online website (https://string-db.org/), the PPI network was analyzed by Cytoscape software, and the top 20 hub genes were calculated using the MCC algorithm of the cytoHubba plugin.

### Hub gene expression and analysis

We used GEPIA online software (http://gepia.cancer-pku.cn/index.html) to analyze the expression of hub genes in OC and normal ovarian tissues; the relationship between the hub gene expression and relapse-free survival (RFS, *n* = 1436) in OC was analyzed using the Kaplan–Meier mapper online website (https://kmplot.com/analysis/), log-rank *p* < 0.05 was considered to be significantly associated [68]. The correlation between hub gene expression and individual cancer stage and patient’s age was analyzed using the TCGA database of the UALCAN online website (http://ualcan.path.uab.edu/) [69].

### Statistical analysis

We used GraphPad Prism software for statistical analysis. The significance of statistical results was assessed by t-test or one-way analysis of variance, where p < 0.05 indicates a significant difference and *p* < 0.01 indicates a very significant difference. All data were shown as mean ± standard deviation.

## Supplementary Information

**Additional file 1: Supplementary Figure 1.** Volcano plot of gene expression in *Wt1* (+KTS)/ *Wt1* (−KTS). (A, B) Volcano plot of gene expression in HOSEpiC; (C, D) Volcano plot of gene expression in IOSE80. Sig: significant; Unsig: unsignificant; Con: control.

## Data Availability

Data generated in this study can be obtained by contacting the corresponding author with their consent.

## Abbreviations

EOC: Epithelial ovarian cancer
*Wt1*: Wilms’ tumor gene
OC: Ovarian cancer
DEGs: Differentially expressed genes
ECM: Extracellular matrix
OCE: Variant cancer epithelium
PPI: Protein–protein interaction
CRC: Colorectal cancer
LIMD2: LIM domain 2
FBS: Fetal bovine serum
RT-qPCR: Real-time quantitative polymerase chain reaction
RFS: Relapse-free survival
