# Supplementary figures and images for: Molecular mechanism of Wilms’ tumor (Wt1) (+/−KTS) variants promoting proliferation and migration of ovarian epithelial cells by bioinformatics analysis
Source: J Ovarian Res. 2023 Feb 24;16:46. doi: 10.1186/s13048-023-01124-2 (PMC9951437; doi:10.1186/s13048-023-01124-2)

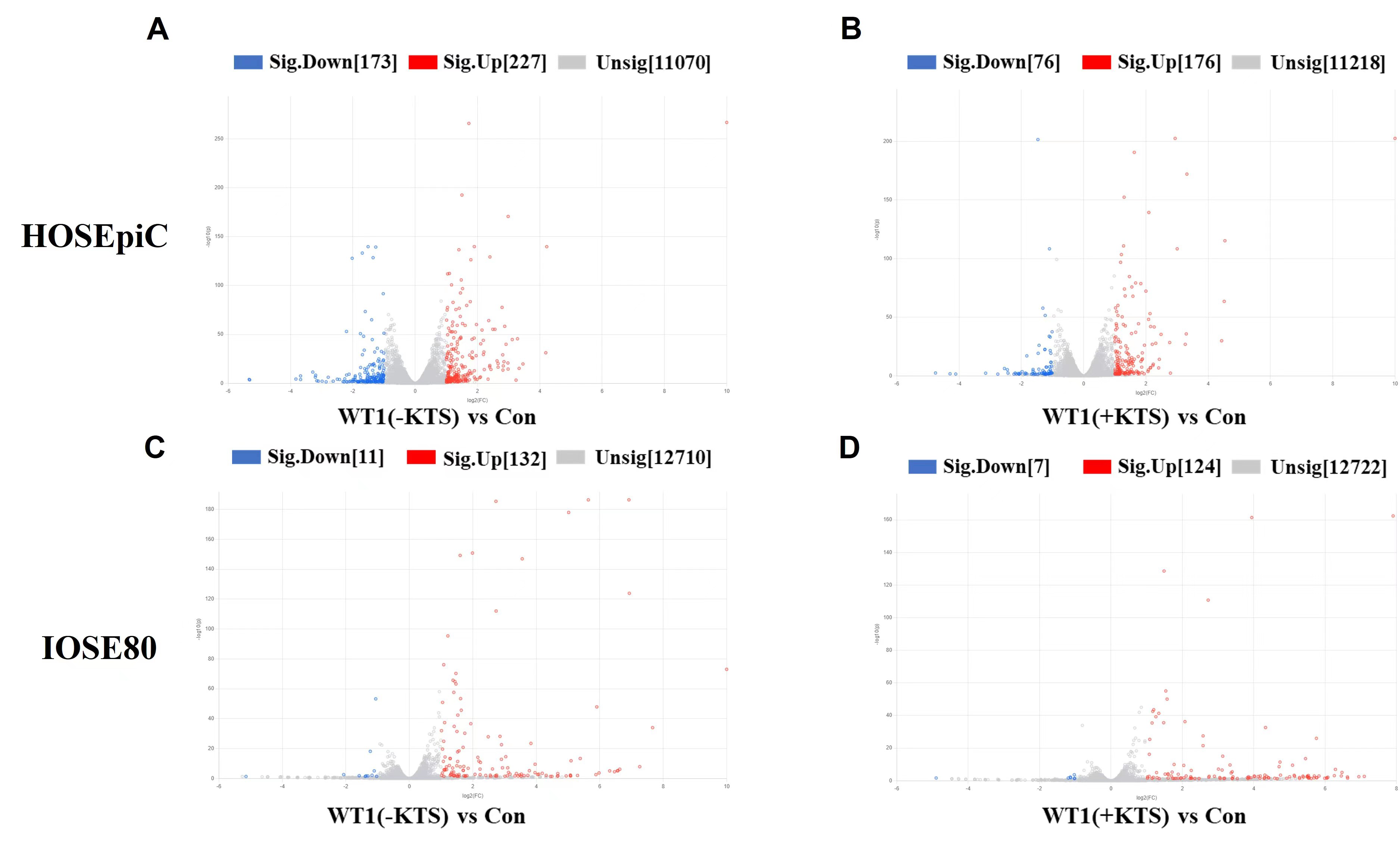

Supplement: Supplementary file 1 — Additional file 1: Supplementary Figure 1. Volcano plot of gene expression in Wt1 (+KTS)/ Wt1 (−KTS). (A, B) Volcano plot of gene expression in HOSEpiC; (C, D) Volcano plot of gene expression in IOSE80. Sig: significant; Unsig: unsignificant; Con: control. [file 13048_2023_1124_MOESM1_ESM.tif]
